# Nasal Myiasis Mimicking Allergic Rhinitis in Immunocompetent Adults: Case Series of 14 Adults

**DOI:** 10.3390/tropicalmed10090257

**Published:** 2025-09-09

**Authors:** Sameh Mezri, Mohamed Anas Ammar, Latifa Mtibaa, Sleheddine Mnasria, Chiraz Halwani, Khemaies Akkari

**Affiliations:** 1 ENT Department, Military Hospital of Tunis, Tunis 1008, Tunisia; 2 Faculty of Medicine of Tunis, University of Tunis El-Manar, Tunis 1068, Tunisia; 3 Laboratory of Parasitology-Mycology, Military Hospital of Tunis, Tunis 1008, Tunisia

**Keywords:** nasal myiasis, *Oestrus ovis*, differential diagnosis, allergic rhinitis, immunocompetent, nasal endoscopy

## Abstract

Background: Human nasal myiasis is a rare zoonotic infection caused by *Oestrus ovis* with a non-specific clinical presentation that can mimic more common conditions such as allergic rhinitis. Objective: To report a series of nasal myiasis cases in immunocompetent individuals, emphasizing the clinical presentation and complementary investigations (endoscopic findings, parasitological identification, skin prick tests, and imaging studies) that facilitate differential diagnosis from allergic rhinitis and enable early treatment. Methods: We conducted a retrospective study including cases of nasal myasis diagnosed and managed at the ENT department of the Military Hospital of Tunis over an 18-year period (2007–2025). Demographic, clinical, diagnostic, and therapeutic data were analyzed. Results: The mean age was 43 years, with a female predominance. Most patients presented with acute rhinological symptoms initially suggestive of allergic rhinitis. Nasal endoscopy revealed larvae in 79% of cases with parasitological confirmation of *Oestrus ovis*. Facial CT scans performed in five cases (36%) were unremarkable. Management consisted of multiple daily nasal saline irrigations and albendazole, in association with oral corticosteroids and antihistamines, resulting in symptom resolution within an average of 4 days. Conclusions: Nasal myiasis should be considered in atypical or treatment-resistant rhinitis. Nasal endoscopy is essential for diagnosis.

## 1. Introduction

Nasal myiasis is a rare zoonotic parasitic infestation (transmitted from animals to humans) caused by fly larvae, primarily *Oestrus ovis*. Humans are accidental hosts, usually after exposure to sheep or goats. Although uncommon [1], its initial clinical presentation can mimic common nasal conditions such as allergic rhinitis, leading to diagnostic delays.

The diagnosis of nasal myiasis relies on the direct visualization of live larvae within the nasal cavity, supplemented by a parasitological examination for precise species identification. Cases in immunocompetent individuals are rare, and no case series have been reported in the literature. To our knowledge, only two individual cases have been described [2,3], highlighting the clinical and epidemiological significance of our series.

In this study, we report a series of 14 cases of nasal myiasis in immunocompetent patients managed at the Military Hospital of Tunis. The aim of our work is to detail the clinical and paraclinical features of this disease, emphasizing the features that allow us to differentiate it from allergic rhinitis, thus ensuring early and appropriate management.

## 2. Methods

We conducted a retrospective study of nasal myiasis cases diagnosed and managed at the ENT Department of the Military Hospital of Tunis. Lavage procedures were performed during consultation, and the recovered fluid was sent for parasitological analysis at the hospital’s parasitology laboratory. Larvae were identified macroscopically and microscopically following Zumpt’s criteria [4]. Identification included evaluation of larval body shape, segmentation, cephalic and posterior spiracular structures, as well as other morphological features enabling the differentiation of *Oestrus ovis* from other fly larvae [4,5].

This study included all patients with nasal myiasis confirmed by endoscopic and/or parasitological examination over an 18-year period (2007–2025).

For each included patient, the following data were extracted from medical records: age, sex, geographical origin (rural/urban), socioeconomic status, presenting symptoms, symptom duration before consultation, initial treatments received, results of clinical and endoscopic examination, results of complementary examinations (allergic skin prick tests, facial CT scans), parasitological identification of the larva, administered treatment, and clinical outcome.

This study complied with the Declaration of Helsinki and was approved by the CLPP of the Tunis Military Hospital (approval no. 81/2024, 22 July 2024).

## 3. Results

We collected fourteen cases of nasal myiasis during the study period. Table 1 summarizes the epidemiological, clinical, diagnostic, and therapeutic data of the cases.

### 3.1. Demographic and Epidemiological Characteristics

The mean age of our patients was 43.2 years (range: 18 to 70 years), with a sex ratio of 0.4 (M/F). Six patients (43%) were from rural areas. Most patients were of medium to high socioeconomic status.

### 3.2. Clinical Presentation and Initial Diagnosis

All patients presented with acute nasal symptoms initially suggestive of allergic rhinitis. Otalgia was reported in three cases (21%). The mean time to consultation was 7.8 days (ranging from 3 to 15 days). All patients had contact with livestock during the ritual slaughter of Eid al-Adha, when animals such as sheep and goats are handled closely, with one patient being a farmer in direct contact with sheep. Initially, all patients were treated empirically with antihistamines and local corticosteroids, with only partial improvement of symptoms. In one case (Case 4 in Table 1), a patient spontaneously expelled a whitish larva during a sneezing effort, prompting him to consult again and guiding the diagnosis.

### 3.3. Ear, Nose, and Throat Examination and Nasal Endoscopy

Upon physical examination, congestion of the vestibule and nasal tip was observed in three patients (21%). All patients had congestive nasal mucosa, and six of them had mucopurulent secretions. Nasal endoscopy systematically revealed the presence of one or more motile, whitish, live larvae circulating within the nasal cavity and reaching the nasopharynx in twelve cases (85.7%), even in the patient who experienced spontaneous expulsion. Two patients had a spontaneous expulsion of the larvae. Acute otitis was diagnosed in two patients.

### 3.4. Complementary Examinations and Parasite Identification

Allergic skin prick tests performed on all patients were negative, thus excluding an underlying allergic rhinitis diagnosis. Facial computed tomography (CT) scans were performed in five patients (36%), revealing no structural abnormalities or signs of infestation extension.

Parasitological examination of the extracted larvae allowed for the identification of *Oestrus ovis* in 10 cases (71%).

Macroscopic and microscopic examination of the larvae enabled us to identify the species *Oestrus ovis* according to Zumpt’s criteria.

**Figure 1:**
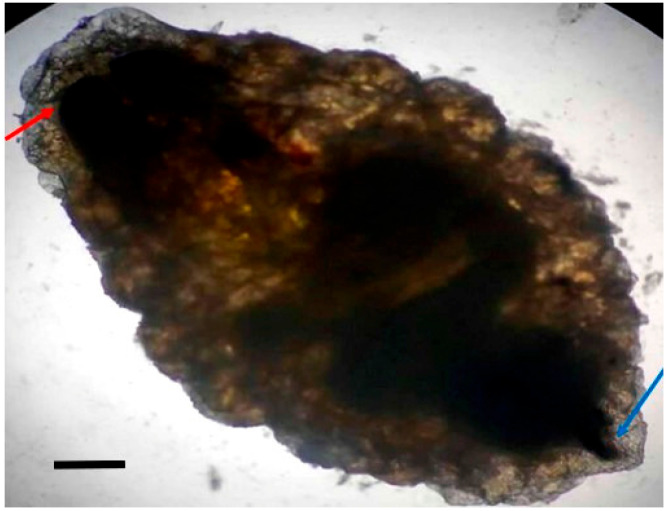
*Oestrus ovis* larva at L2 stage (6 mm) (red arrow: posterior extremity; blue arrow: anterior extremity (Case 4)) observed under microscope (objective ×4; scale bar = 1 mm).

**Figure 2:**
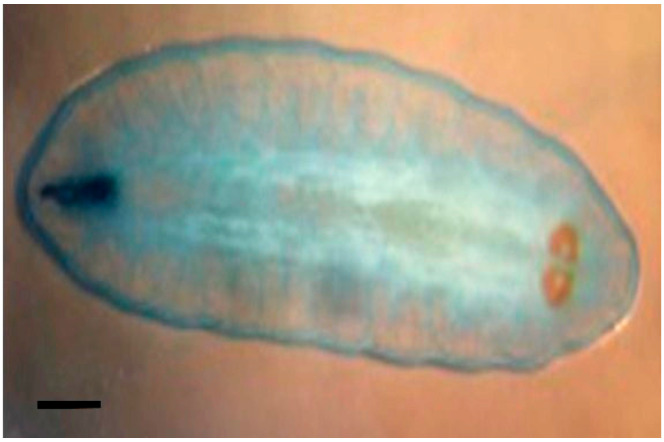
*Oestrus ovis* larva at L2 stage (8 mm) (Case 5) observed under microscope (objective ×4;scale bar = 1 mm).

**Figure 3:**
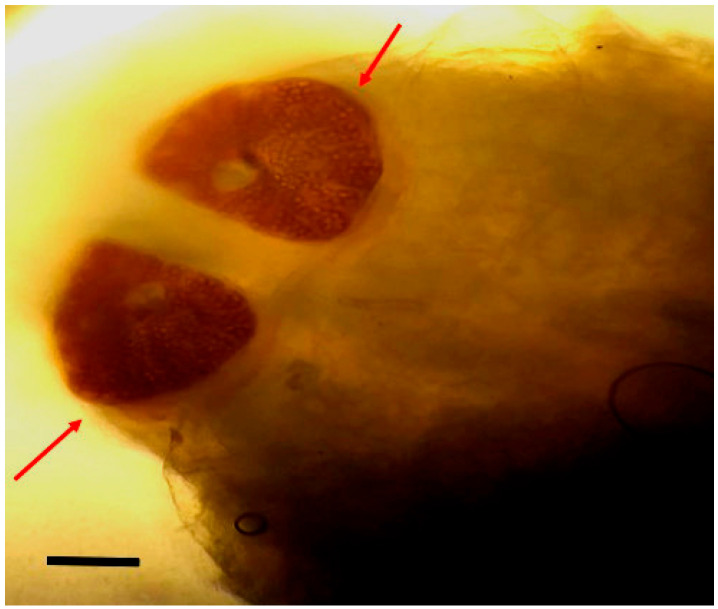
Posterior extremity of *Oestrus ovis* larva showing respiratory stigmata (red arrows) (scale bar = 100 µm) (Case 4).

**Figure 4:**
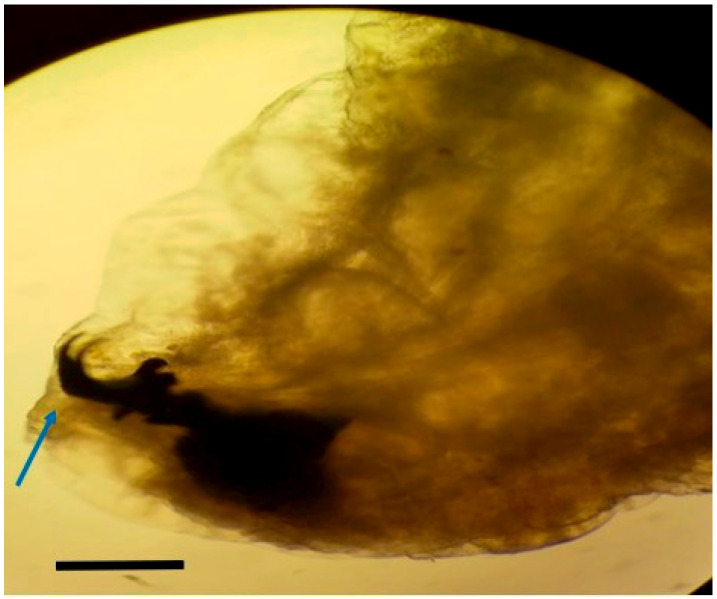
Anterior extremity of *Oestrus ovis* larva showing mouth hooks (blue arrow) (scale bar = 100 µm) (Case 4).

Larvae were identified as *Oestrus ovis* at the L2 stage (5–6 mm). They were semi-cylindrical, with posterior subcircular respiratory stigmata and a pseudocephalon bearing two buccal hooks (Figure 1, Figure 2, Figure 3 and Figure 4).

Species identification could not be achieved in four cases because of inadequate specimen preservation following home collection.

### 3.5. Treatment and Outcome

Treatment involved multiple daily irrigations with 500 mL of normal saline solution (0.9%) containing 400 mg of albendazole, administered for 4 to 7 days. All patients also received intranasal corticosteroids and oral antihistamines. In cases complicated by bacterial superinfection, antibiotic therapy was added in combination with systemic corticosteroids.

Clinical improvement was rapidly achieved, with symptom resolution in an average of 4 days. No severe complications or recurrences were observed during follow-up.

## 4. Discussion

This case series highlights the diagnostic challenges of nasal myiasis in immunocompetent patients. All cases were initially misdiagnosed as allergic rhinitis, underlining the need for awareness among clinicians in endemic regions.

### 4.1. Diagnostic Challenges

The initial symptoms reported by patients are non-specific. Intense nasal pruritus, sudden-onset sneezing, nasal obstruction, rhinorrhea, and headache closely resemble acute allergic rhinitis. This overlap, together with the rarity of nasal myiasis, likely contributes to delayed diagnosis and ineffective initial treatment with antihistamines and topical corticosteroids. Although allergic rhinitis is common, our findings emphasize the need for increased clinical suspicion when symptoms are atypical, severe, or treatment-resistant [5,6]. Sensations of movement in the nose, severe pain, or epistaxis are “red flags” that help distinguish myiasis [7]. In our series, spontaneous expulsion of a larva during a sneeze provided an immediate diagnostic clue.

Certain features should prompt further investigation. Otalgia, observed in three cases, is unusual but notable. Sudden onset, lack of response to standard anti-allergic therapy, and mucopurulent secretions were consistent indicators of nasal myiasis, possibly reflecting larval migration or local inflammation. The diagnosis was confirmed parasitologically by macroscopic and microscopic identification of the expelled larvae according to established morphological criteria [5], ensuring accurate differentiation from other dipteran species.

Nasal endoscopy is essential as it can promptly reveal the characteristic whitish, motile larvae, even when symptoms are mild, supporting recommendations for rapid endoscopic evaluation in atypical or persistent cases, especially after treatment failure [8]. Negative allergological skin tests further exclude allergic rhinitis.

CT scans were normal in all patients, confirming the limited role of imaging in early disease and reinforcing the importance of nasal endoscopy for timely diagnosis.

### 4.2. Epidemiology

Our series, with 43% of patients originating from rural areas, aligns with the known epidemiology of *Oestrus ovis* myiasis, which is primarily a disease of sheep and goats, with humans becoming accidental hosts, often through direct contact or proximity to infested animals [9]. The presence of myiasis in individuals with a “medium to high socioeconomic level” and who are immunocompetent is particularly interesting. While myiasis is often associated with poor hygiene, debility, or immunocompromised states [10,11], our cases—where all patients had contact with livestock (sheep or goats) during the ritual slaughter of Eid al-Adha—indicate that even otherwise healthy individuals can be at risk in the appropriate epidemiological context. This highlights that myiasis should not be dismissed based solely on patient demographics or general health status, as noted by Ravichandran [2] and Einer [3]. Ravichandran K [2] recently reported a case of nasal myasis in a 40-year-old immunocompetent patient with no predictive factors consulting for nasal bleeding.

### 4.3. Treatment

The therapeutic management of nasal myasis remains debated due to the rarity of the condition and the absence of standardized protocols. Reported strategies generally combine mechanical removal or induction of larval expulsion with antiparasitic/irritant agents, complemented by anti-inflammatory measures: endoscopic extraction is often challenging because of larval mobility, hence the frequent use of irritating or asphyxiating substances to facilitate elimination [12].

Adjunctive therapies have been described, particularly in severe or immunocompromised patients, including antiparasitic agents such as ivermectin, antibiotics such as clindamycin, and irritants like turpentine oil [13] with good evolution. Koray K [14] reported complete recovery in two immunocompromised patients managed with mechanical debridement plus diluted povidone–iodine and saline, while advising against systemic antiparasitic or antibiotic therapy to limit the pharmacologic burden. Conversely, Camron D [15] observed favorable outcomes in five immunocompromised (intubated) patients treated with systemic and topical ivermectin, achieving recovery within seven days.

In an immunocompetent patient, Ravichandran K [2] reported successful outcomes with endoscopic manual extraction aided by turpentine oil, followed by saline irrigation and local wound surgical debridement. Collectively, these reports underscore the heterogeneity of approaches and the lack of consensus.

Because cases are scarce, especially in immunocompetent individuals, meaningful comparison of treatment safety and efficacity is not feasible, as each team has applied its own protocol. Notably, we have found no published use of albendazole for nasal myasis.

In our series, as all patients were immunocompetent, we adopted a topical regimen consisting of saline nasal lavages mixed with albendazole (the only locally available topical anthelmintic in Tunisia), combined with corticosteroids and antihistamines. This approach yielded rapid clinical improvement within an average of four days, with no recurrences or complications on follow-up, highlighting a practical, safe, and cost-effective option in resource-limited settings.

### 4.4. Limitations

Our study’s main limitation is its retrospective nature and the relatively small sample size of fourteen cases. Although invaluable for a rare disease, this limits the generalizability of our findings. Furthermore, precise larval staging and complete species identification were not possible in three cases.

## 5. Conclusions

This case series highlights nasal myiasis as an important differential diagnosis in patients with atypical or treatment-resistant rhinitis, even in immunocompetent individuals. Clinicians in endemic regions should consider nasal myiasis in refractory rhinitis, utilizing endoscopy for early diagnosis and treatment.

## Figures and Tables

**Table 1:** Clinical, diagnostic, epidemiological, and therapeutic features of 14 nasal myiasis cases at the Otorhinolaryngology Department, Military Hospital of Tunis (2007–2025).

Patients	Age Years	Gender	Antecedents *	Origin	Job	Symptoms	Delay to Consult	Initial Treatment	Examination	Larva Identification
1	24	F	0	U	Engineer	Pruritus Rhinorrhea	3 days	Irrigation ** Corticoid Anti histamine	Larva moving	*Oestrus ovis*
2	69	F	Gastric ulcer	U	Homemaker	Pruritus	6 days	Irrigation ** Antibiotic	Larva moving	*Oestrus ovis*
3	52	F	HTN	R	Factory	Rhinorrhea larva expulsion pharyngitis	6 days	Irrigation ** Antihistamine	Larva moving	*Oestrus ovis*
4	38	F	Anti-synthetase Sd Immunosuppressive therapy Corticoids.	U	Airport	Rhinorrhea larva expulsion	3 days	Irrigation **	Congestive rhinitis	*Oestrus ovis*
5	50	F	0		Airport	Pharyngitis rhinorrhea Larva expulsion	10 days	Irrigation **	Rhino-pharyngitis	*Oestrus ovis*
6	43	H	0	U	Company manager	Nasal obstruction Pruritus	15 days	Irrigation ** Antihistamine	Larva moving	*Oestrus ovis*
7	24	H	0	R	Student	Nasal obstruction Pruritus	10 days	Irrigation ** Antibiotic corticoid Antihistamine	Larva moving	-
**8**	70	F	HTN Diabetes	R	Homemaker	Nasal Pharyngeal Pruritus	6 days	Irrigation ** Antihistamine	Larva moving	-
**9**	57	F	0	R	Homemaker	Nasal pruritus	11 days	Irrigation **	Larva moving	*Oestrus ovis*
**10**	44	F	0	R	Homemaker	Pruritus	5 days	Irrigation ** antihistamine	Larva moving	*Oestrus ovis*
**11**	18	F	0	U	Student	Pruritus Otitis	8 days	Irrigation ** Antibiotic Corticoid Antihistamine	Larva moving	*Oestrus ovis*
**12**	29	H	0	U	Restaurant agent	Pruritus Otitis Sinusitis	6 days	Irrigation ** Corticoid Antibiotic	Larva moving	*Oestrus ovis*
**13**	52	H	0	R	Farmer	Nasal obstruction Pruritus	7 days	Irrigation ** Corticoid Antibiotic Antihistamine	Larva moving	-
**14**	36	F	0	U	Aesthetician	Pruritus Hyposmia	15 days	Irrigation ** Corticoid Antibiotic	Larva moving	-

F = female; M = male; U = urban; R = rural; HTN = arterial hypertension; * 0 = presumably “none”; Irrigation ** = 500 mL of saline solution with 400 mg of albendazole.

## Data Availability

The original contributions presented in the study are included in the article, further inquiries can be directed to the corresponding author.

## References

[1] Delhaes L., Bourel B., Pinatel F., Cailliez J.C., Gosset D., Camus D., Dei-Cas E. (2001). Myiase nasale humaine à Œstrus ovis [Human nasal myiasis due to Oestrus ovis]. Parasite.

[2] Ravichandran K., Padmanabhan K., Thomas S.K., S D.P., Liji N. (2025). Unusual Presentation of Nasal Myiasis in Immunocompetent Young Individual: Case Report. Indian J. Otolaryngol. Head Neck Surg..

[3] Einer H., Ellegård E. (2011). Nasal myiasis by Oestrus ovis second stage larva in an immunocompetent man: Case report and literature review. J. Laryngol. Otol..

[4] Zumpt F. (1965). Myiasis in Man and Animals in the Old World: A Textbook for Physicians, Veterinarians and Zoologists.

[5] Martínez-Calabuig N., Panadero R., Varaz G., Remesar S., Lopez C.L., Saldaña A., Diaz P., Diez-Banos P., Morrrondo P., Garcia-Dios D. (2024). Application of Zumpt’s criteria for larval identification of Oestrus ovis and Cephenemyia stimulator in cervid hosts. Eur. J. Wildl. Res..

[6] Sante Fernández L., Hernández-Porto M., Tinguaro V., Lecuona-Fernández M. (2017). Ophthalmomyiasis and nasal myiasis by Oestrus ovis in a patient from the Canary Islands with uncommon epidemiological characteristics. Enfermedades Infecc. Microbiol. Clin..

[7] Gupta S.K., Nema H.V. (1997). Rhino-orbital-myiasis. J. Laryngol. Otol..

[8] Soni N.K. (2000). Endoscopy in nasal myiasis. Trop. Doct..

[9] Tyagi A.K., Suji P.S., Kumar A., Varshney S., Mohanty A., Gupta P. (2022). First Report on Concomitant Infection of Nasal Myiasis and Trichosporonosis in an Uncontrolled Diabetic Patient: Case Report. Indian J. Otolaryngol. Head Neck Surg..

[10] Savaş N., Aykur M. (2024). Oral and Nasal Myiasis in Two Patients Hospitalized in the Intensive Care Unit: Diagnosis and Clinical Significance of Cases. Indian J. Otolaryngol. Head Neck Surg..

[11] Francesconi F., Lupi O. (2012). Myiasis. Clin. Microbiol. Rev..

[12] Sayeed A., Ahmed A., Sharma S.C., Hasan S.A. (2019). Ivermectin: A Novel Method of Treatment of Nasal and Nasopharyngeal Myiasis. Indian J. Otolaryngol. Head Neck Surg..

[13] Singh T.K., Sankar H., Gupta A., Kumar M. (2023). Oral myiasis in an immunocompromised adult undergoing chemotherapy: A rare case and comprehensive treatment protocol. Cureus.

[14] Koray K. (2025). Oral and Paranasal Myiasis: Two Case Reports. SN Compr. Clin. Med..

[15] Camron D., Tate N., Sanjeet R., Robert Y. (2023). Combination ivermectin therapy to treat nasal myiasis: A case series. Otolaryngol. Case Rep..

